# On the Causes of Dental Caries

**Published:** 1882-09

**Authors:** J. R. Mummery

**Affiliations:** London


// THE DENTAL REGISTER.

VOL. XXXVI.] SEPTEMBER, 1882 [No. 9.

### ON THE CAUSES OF DENTAL CARIES.

Mr. J. R. MUMMERY, London.

[Read before the International Medical Congress, London, August, 1881.]

The question is perpetually recurring.—To what causes are we to attribute the admitted degeneracy of the teeth, and contraction of the maxillary arch, at the present day? Many theories have been advanced upon the subject, but a sufficiently extended and systematic accumulation of reliable data, remains still a desideratum. Nearly twenty years have passed away since I commenced a series of investigations into the conditions of the teeth and maxilla of the races who have successively inhabited this country, and of the ruder existing races of mankind.

In pursuance of this object I visited, in the course of nine years, every known collection of skulls, between the museums of Netley Hospital, in the south, and that of Newcastle, in the north.

More than 3,000 skulls passed through my hands, but a large proportion were without a reliable history; I selected nearly 2,000 and recorded the results of a careful examination in a tabular form; appending such information as could be attained from their cemeteries, their weapons, and from historical notices, with reference to the food and social advancement of ancient races in England.

With regard to existing uncivilized races, I have derived much valuable information from military and other gentlemen who had resided in, or had traveled through, countries inhabited by half savage tribes.

In the year 1869 I had the honor of reading a paper upon the subject, at a meeting of the Odontological Society of Great Britain.

With regard to the ancient inhabitants of this country, I found a very striking infrequency of caries, and no instance of irregularity, among the earliest races (whether Palæolithic or Neolithic), who were unacquainted with the use of metals. A marked increase is observable in the tendency to caries in the Celtic race who succeeded them, and whose possession of bronze instruments, and considerable progress in civilization, is proved by evidence from many sources.

After the Roman conquest, the instances of extensive caries and irregularities, arising from contracted jaws, are very frequent, bearing the surprising proportion of ten cases of caries to one case found among the earliest race of people; and we have abundant historic proof of the luxurious habits and advanced mental culture introduced by the conquerors.

The influx of the mighty hordes of Teutonic race, or half savage people of simple habits of life, was followed by a very remarkable diminution in the number and severity of cases of caries, and irregularity of the teeth.

It is generally admitted that civilized life, with its high mental culture, has a tendency to stimulate the development of the brain, whilst the maxillary bones, in the absence of the severe employment of the teeth, in savage conditions of life, suffer a proportionate diminution in size and power.

It should, however be noticed that destructive attrition of the grinding surfaces of the teeth is singularly prevalent among people, whether in ancient or modern times, whose food is imperfectly cooked, and, consequently, tough or hard, when compared with people of civilized habits.

It would be out of place to occupy the Section by further reference to a paper road some time ago; but it appeared necessary to make a brief mention of my too limited investigations by way of introduction.

The gathering of such an unprecedented number of scientific men at this great Congress, has afforded me an opportunity of enlisting efficient aid in research which should not be lost, and I offer the following list of questions, proposed for distribution by authority of the Congress:

1. Have you any opportunity of comparing the teeth of mountain dwellers with those of a kindred race of people who inhabit marshy plains or insalubrious valleys?

2. Have you observed any injurious effects upon the teeth attributable to the impregnation of drinking water with sulphurous acid gas, in volcanic or in coal-mining districts?

3. Have you any facilities for comparing the condition of the teeth among factory operatives, with those of an agricultural population in a neighbouring district?

4. Have you an opportunity of noticing any especially healthy state of the teeth, and a fuller development of the maxilla, among sailors, whose diet of hard biscuits and tough meat demands efficient mastication, thus of necessity approaching the habits of uncivilized races, and similarly experiencing destructive attrition?

5. Have you observed, among communities in a similar rank of life, who subsist on a mixed, and often unwholesome diet, that requires but little mastication, a less favorable condition of the jawbones and teeth?

6. Have you seen any remarkably healthy state of the dental organs among people who subsist upon oatmeal, pure wheat meal. maize, or leguminous food, as compared with those living upon potatoes, or other food, deficient in albuminoid elements?

7. Are you of opinion that the frequent sucking of sweets, especially when combined with citric or other acids, may be regarded as one cause of the increasing prevalence of dental caries?

8 Have you observed any instances of the alleged injurious effect of camphor as an ingredient in tooth-powder?

9. In instances of the emigration of families, who, quitting a highly artificial state of life, have settled in a healthy district or country, adopting simple habits and diet; have you in such cases had the opportunity of comparing the diseased state of the teeth in the elder children with those of the children born under the later and more salubrious conditions?

10. Have you observed in certain families, who, for a long series of years, have been under your professional care, a progressive deterioration of the teeth, in each succeeding generation?

11. Have you known instances in which the cumulative influence of hereditary disease consequent upon repeated intermarriages, has manifested itself in contracted maxillary arch and extensive dental caries?

12. Have any cases come under your notice which led you to conclude that injury to the teeth may sometimes be traceable to the over-tasking the intellect of young children, seeing that the brain is undergoing its most rapid increase in volume at the precise period of life in which the whole of the permanent teeth, with the exception of the third molars, are in process of development?

